# Feeding practices and early weaning in the neonatal period: a cohort study

**DOI:** 10.11606/s1518-8787.2021055003248

**Published:** 2021-10-13

**Authors:** Josilene Maria Ferreira Pinheiro, Taiana Brito Menêzes Flor, Mayara Gabrielly Germano de Araújo, Ana Márcia Soares Fernandes Xavier, Amanda Michelly Braga da Mata, Vanessa Cristina da Costa Pires, Luana Isabelly Carneiro de Oliveira, Fábia Barbosa de Andrade

**Affiliations:** I Universidade Federal do Rio Grande do Norte Programa de Pós-Graduação em Saúde Coletiva NatalRN Brasil Universidade Federal do Rio Grande do Norte. Programa de Pós-Graduação em Saúde Coletiva. Natal, RN, Brasil; II Universidade Federal do Rio Grande do Norte Hospital Universitário Onofre Lopes NatalRN Brasil Universidade Federal do Rio Grande do Norte. Hospital Universitário Onofre Lopes. Natal, RN, Brasil; III Universidade Federal do Rio Grande do Norte NatalRN Brasil Universidade Federal do Rio Grande do Norte. Curso de Graduação em Nutrição. Natal, RN, Brasil

**Keywords:** Breast Feeding, Weaning, Mixed Feeding, Infant Formula, Infant Nutrition, Cohort Studies

## Abstract

**OBJECTIVE:**

To describe feeding practices and the risk factors for the mixed breastfeeding and early weaning in the neonatal period.

**METHODS:**

Cohort study, which we collected socioeconomic, demographic, health care and feeding data from 415 mother/child binomials born in four public maternity hospitals in Natal/Brazil. They were followed-up at 48 hours, 7 and 28 days after birth. The association was established using Pearson’s Chi-square test and Poisson’s regression, after adjusting it to other variables.

**RESULTS:**

The prevalence of mixed breastfeeding in the first 2 days was 47,2% and early weaning in 7 and 28 days was 8,4% and 16,2% in that order. The main reasons for mixed breastfeeding and early weaning were: colostrum deficiency (33.8%), difficulty in latching/sucking (23.5%) and “little milk” (70.0%). The use of formula/milk/porridge remained associated with maternal age ≤ 20 years (RR = 0.64; 95%CI: 0.47–0.86), age 20–29 years (RR = 0,70; 95%CI: 0,57–0,87), primiparity (RR = 1.37; 95%CI: 1.11–1.60) and cesarean delivery (RR = 1.20; 95%CI: 1.00–1.45) at 2 days; absence of paternal support (RR = 4.98; 95%CI: 2.54–9.79) and pacifier use (RR = 3.21; 95%CI: 1.63–6.32) at 7 days; and only pacifier use (RR = 2.48; 95%CI: 1.53–4.02) at 28 days.

**CONCLUSIONS:**

Early weaning was associated with maternal and health care factors, thus suggesting the need to readjust good practices and educational actions to achieve the exclusive offer to the maternal breast in the neonatal period.

## INTRODUCTION

In the neonatal period (0 to 28 days), there is great biological and social vulnerability. For this reason, feeding has a fundamental effect on the child’s nutritional status, growth and development^1^. From this perspective, feeding practices, which are influenced by social, cultural, and economic issues, are based on the knowledge of healthy food choices, preparation methods, time, opportune moment, environment, frequency and how to offer food to the newborn^2^.

Exclusive breastfeeding (EBF) is the best food to be offered to the newborn in the neonatal period; and, when extended to 6 months, it is associated with suitable nutritional status, reduction of infections and lower mortality. Despite the nutritional and immunological superiority of breast milk over other types of milk, the prevalence of approximately 38% of EBF in the first 6 months of life is below the rate recommended by the World Health Organization (WHO). Although the prevalence of this practice has grown in recent decades, there is a trend toward stabilization in many countries^3^. These data are targets of the WHO, which plans to raise these rates to 50% by the year 2025.^3^

In Brazil, the results of *Estudo Nacional de Alimentação e Nutrição Infantil* (ENANI – National Study of Child Food and Nutrition) in 2019, carried out in all regions of the country from February 2019 to March 2020, show the prevalence of 60% EBF for children under 4 months and 45.7% for children under 6 months. Nevertheless, such indicators are still unevenly distributed in different regions. Their lower prevalence is in the northeast region, with rates of 55.8% under 4 months and 33.8% under 6 months^6^.

The same is observed for the act of breastfeeding in the first hour of life – considered an indicator that favors prolonged breastfeeding – and, subsequently, in the first 28 days^7^. Nevertheless, it remains considerably less than 80%, the minimum rate recommended by the Baby-Friendly Hospital Initiative (BFHI)^8^.

In spite of the existence of measures to protect breastfeeding, early weaning is still a reality. The use of infant formula while still in the maternity hospital – whether due to justifiable indications by the BFHI or not – has been increasingly used and seems to be associated with it^9,10^. This practice is also related with infections, malnutrition, excessive weight gain, food allergy, diabetes and the use of pacifier and baby bottle^5,7,11^.

Although the pertinent literature explains the determinants of early weaning, there is a lack of studies that address the feeding practices in the first 28 days of life of the newborn. Thus, we aimed to describe feeding practices and risk factors for the mixed breastfeeding and early weaning in the neonatal period.

## METHODS

### Design and Setting

It was a prospective cohort study conducted from February to August 2019 with mother/newborn binomials cared for in four public maternity hospital in Natal (Rio Grande do Norte/Brazil). This research is part of a larger project, which is assessing the health care actions targeted at the neonate, recommended by the Brazilian Ministry of Health (MH).

### Data Collection

In order to perform the sample calculation, we considered the Neonatal Call/2010 survey to 70% prevalence for EBF^13^, a sample error of 5% for a population of 14,025 live births in the year 2018, confidence level of 95% and non-response rate of 24%, thus determining a sample of 415 mother/child binomials. The sample was stratified according to the number of live births in each of the maternity hospital in the year 2018, whose distribution was as follows: 31% in maternity A (129 mother/child pairs); 21% in maternity B (88 mother/child pairs); 23% in maternity C (96 mother/child pairs) and 25% in maternity D (102 mother/child pairs).

We included full-term newborns, from single pregnancy, aged ≥ 37 weeks, with birth weight ≥ 2,500g and Apgar ≥ 7 in the 1^st^ and 5^th^ minutes. We excluded those newborns with congenital malformation and that needed intensive care and whose mothers were unable to answer the questionnaire because her general health conditions.

The data were collected by means of a questionnaire previously prepared and tested by the research team. They were previously trained in a pilot study with 30 binomials in maternity hospital A, elected for convenience.

The data were collected at 48 hours after birth in the morning and afternoon shifts every day of the week until the stipulated sample size for each maternity hospital was reached. The binomial data were obtained from the medical records; when some information was absent, the pregnant woman’s card and, ultimately, the mother’s reports were considered. In the second and third collection periods (at 7 and 28 days, respectively), the research occurred through telephone calls. Losses of follow-up were considered after three unsuccessful telephone contact attempts.

### Data Measures

We considered as “outcome” the variables mixed breastfeeding offered to newborn in the maternity hospitals and early weaning at home after hospital discharge. In addition, EBF was defined when the mother exclusively breastfed or offered breast milk from milk banks. There was mixed breastfeeding when breast milk was complemented with starting infant formula and early weaning when breast milk was substituted by infant formula or cow’s milk or porridge (cow’s milk or formula, cereal and sugar). We classified the dependent variables as EBF (score = 0) and mixed breastfeeding and early weaning (score = 1). The mother was also asked about the supply of water and tea. Predominant breastfeeding was defined as the condition in which mothers offer breastfeeding and other liquids based on water. In this study, there was no predominant breastfeeding. The characterization of the food offered to newborn included the volume, the suitability of the infant formula dilution or cow’s milk, the reasons for offering the latter two and utensils used to administer.

In order to explain the outcome of the food offered to newborn in the neonatal period, we considered maternal variables regarding socioeconomic status: age (< 20 years, 20 to 29 years, ≥ 30 years), education (High School /Higher Education Elementary School), income (> 1 minimum salary, ≤ 1 minimum salary), marital status (Single/widowed/divorced, Married/stable relationship) and occupation (Student, Housewife, Grower, Works outside the home). The individual characteristics were categorized as: parity (Multiparous and Primiparous), method of delivery (Vaginal and Cesarean Section) and having diabetes (Yes or No). We also assessed the individual variables of newborn: gender (Female and Male), the birth weight (2,500g to 4,000g and > 4,000g) and the color variable was self-declared by the mother (White, Brown, Black). The other variables were categorized as Yes and No. The care variables were included: guidelines on breastfeeding during prenatal care and during hospitalization, companion during delivery, skin-to-skin contact, breastfeeding in the first hour, home visit, paternal support to breastfeed, the offer of pacifier, baby bottle and the offer of complement to breastfeeding in the first 48 hours). The categories being coded in 0 and in 1 (risk).

### Statistical Analysis

All statistical analyzes were conducted by means of SPSS® software, version 20.0. In order to check the differences between the individual characteristics of the binomial and the initial associations, we used Pearson’s Chi-square test, with 95% confidence intervals. We used Poisson’s regression to establish the strength of the association, with Relative Risk (RR), between the predisposing factors and the outcome. Only variables with p value ≤ 0.20 in Pearson’s Chi-square were included in the multiple Poisson’s model, removing variables with p value > 0.10. Variables with significant p ≤ 0.05 remained in the final model. The analyzes met the assumptions established for the adjustment (Goodness off fit), significance (Omnibus Test) and dispersion.

### Ethics

The study was approved by the Research Ethics Committee of the *Hospital Universitário Onofre Lopes* (Natal, Brazil), under opinion nº 3.133217, being in line with Resolution nº 466/2012. The parents signed an informed consent for participating in the study.

## RESULTS

In the neonatal period, 415 mother/child binomials were initially recruited. In the first week, this number decreased to 358 (loss of 13.7%); and, at the end of the 28 days, to 346 (loss of 16.6%). The surveyed population (mothers and newborns) was similar among the study settings. Most mothers were in the age group from 20 to 29 years old (46.5%), had high school/higher education (65.3%), were married or lived in a stable relationship (79%), had an income ≤ 1 minimum wage (64.7%) and were multiparous (62.1%). As for the newborns, 52.5% were male and 92.1% weighed among 2500g and 4000g (Table 1).

**Table 1:** Description of the characteristics of mothers and newborns assisted in public maternity hospitals in the city of Natal/Brazil, 2019 (n = 415).

Variables	Maternity hospital					p^a^

	Total	A	B	C	D	
Maternal characteristics						
Age						0.081
< 20 years	79 (19.0)	20 (15.5)	17 (19.3)	20 (20.8)	22 (21.6)	
20 to 29 years	193 (46.5)	52 (40.3)	48 (54.5)	41 (42.7)	52 (51.0)	
≥ 30 years	143 (34.5)	57 (44.2)	23 (26.1)	35 (36.5)	28 (27.5)	
Educational level						0.639
High School/Higher Education	271 (65.3)	87 (67.4)	55 (62.5)	59 (61.5)	70 (68.6)	
Elementary school	144 (34.7)	42 (32.6)	33 (37.5)	37 (38.5)	32 (31.4)	
Marital status						0.236
Single/widowed/divorced	87 (21.0)	22 (17.1)	25 (28.4)	20 (20.8)	20 (19.6)	
Married/stable relationship	328(79.0)	107 (82.9)	63 (71.6)	82 (79.2)	82 (80.4)	
Occupation						0.501
Student	44 (11.5)	12 (10.0)	10 (13.5)	14 (15.2)	8 (8.2)	
Housewife	223 (58.1)	70 (58.3)	41 (55.4)	54 (58.7)	58 (59.2)	
Grower	93 (24.2)	13 (10.8)	0 (0.0)	10 (10.9)	1 (1.0)	
Works outside the home	24 (6.3)	25 (20.8)	23 (31.1)	14 (15.1)	31 (31.6)	
Family income						0.009
> 1 minimum salary	147 (35.4)	47 (36.4)	34 (38.6)	21 (21.9)	45 (44.1)	
≤ 1 minimum salary	268 (64.6)	82 (63.6)	54 (61.4)	75 (78.1)	57 (55.9)	
Parity						0.067
Primiparous	154 (37.1)	37 (28.7)	38 (43.2)	42 (43.8)	37 (36.3)	
Multiparous	261 (62.9)	92 (71.3)	50 (56.8)	54 (56.8)	65 (63.7)	
Newborn’s characteristics						
Gender						0.290
Female	197 (47.5)	64 (49.6)	45 (51.1)	48 (50.0)	40.0 (39.2)	
Male	218 (52.5)	65 (50.4)	43 (48.9)	48 (50.0)	62.0 (60.8)	
Color						0.732
White	137 (33.0)	41 (31.8)	33 (37.5)	27 (28.1)	36 (35.3)	
Brown	274 (66.0)	86 (66.7)	54 (61.4)	68 (70.8)	66 (64.7)	
Black	4 (1.0)	2 (1.6)	1 (1.1)	1 (1.0)	0 (00.0)	
Birth weight						0.272
2,500–4,000g	375 (92.1)	121 (96.0)	80 (90.9)	86 (90.5)	88 (89.8)	
> 4,000g	32 (7.9)	5 (4.0)	8 (9.1)	9 (9.5)	10 (10.2)	

^a^ Chi-square p-value.

Table 2 shows the evolution of the feeding of newborn in the neonatal period. In the first 2 days, the EBF rate was 48.7%, evolving to 91.6% at 7 days, followed by a slight reduction at the end of the first month (83.8%). Regarding to mixed breastfeeding rate, it was found out 47.2% in first 2 days. Early weaning increased along the follow up, reaching 16.2% at the end of 28 days. The use of infant formula was lower in the first week of life, followed by a small increase at the end of the neonatal period. The porridge was offered for a little more than 1% of newborn at 7 and 28 days; while the water, for 14.2% of newborn after the first week; and the tea was not offered at any time.

**Table 2:** Mixed breastfeeding and early weaning: characteristics and reasons for offering the food and in the neonatal period. Natal/Brazil, 2019.

Variables	2 days	7 days	28 days
		
	n (%)	n (%)	n (%)
	n = 415	n = 358	n = 346
Mixed breast feeding	196 (47,2)	-	-
Early weaning	-	30 (8,4)	56 (16,2)
			
Type of food offered to newborn			
Exclusive breast milk	202 (48,7)	328 (91,6)	290 (83,8)
Pasteurized breast milk	17 (4,1)	-	-
Infant formula	196 (47,2)	26 (7,3)	49 (14,2)
Porridge^a^	-	4 (1,1)	7 (2,0)
Tea	-	-	-
Water	-	-	49 (14,2)

Reasons for offering the complement/substitute	n = 213	n = 30	n = 56
Colostrum deficiency	72 (33.8)	-	-
Difficulty in latching/sucking	50 (23.5)	-	-
Little milk	-	21 (70.0)	43 (76.8)
Weak milk	-	01 (3.3)	
LGA	07 (3.3)		
Child’s refusal	-	-	03 (5.4)
Other problems related to newborn	18 (8.4)	03 (10.0)	-
Breast problems	06 (2.8)	02 (6.7)	03 (5.4)
Other problems related to the mother	09 (4.2)	02 (6.7)	-
Doctor’s prescription	-	-	07 (12.5)
Without prescribed/expressed reasons	51 (23.9)	01 (3.3)	-

Dilution of infant formula/milk^b^			
Suitable	213 (100.0)	16 (53.3)	25 (44.6)
Not suitable	-	14 (46.7)	31 (55.4)

Utensils used to administer			
baby bottle	-	30 (100.0)	55 (85.9)
Cup	213 (100.0)	-	09 (14.1)
Volume offered in mL (average and SD)	11.4 (4.0)	42.3 (20.1)	75 (26.0)

LGA: large for gestational age.

^a^ Porridge: preparation made with infant formula/cow’s milk + cereal and sugar.

^b^ Dilution of infant formula/milk: dilution according to manufacturer’s guidelines.

The reasons for offering complement or substitute to breast milk are expressed in Table 2. The colostrum deficiency in the first 2 days (33.8%) – reported by the health care team – and the “little milk” at 7 (70.0%) and 28 days (76.8%) – reported by the mothers – were the main claims. Infant formula was the most offered complement throughout the period, whose average volume was progressive. It increased from 11.4 mL to 42.3 mL and, subsequently, 75 mL. In the maternity hospitals, the complement was offered by means of cup; and, at home, by baby bottle and cup. When the dilution of the complement was assessed, we found that it was suitable, in its totality, when performed in the maternity hospitals.

After hospital discharge, when diluted by the mothers, only 53.3% and 44.6% did it correctly at 7 and 28 days, in that order. Although health care practices related to the guidance and encouragement of EBF (such as guidelines on breastfeeding during prenatal and hospitalization, skin-to-skin contact, guidance on breast care and on the non-use of pacifier and baby bottle, and home visits) had been performed (Table 3), these variables were not significant as protection factors to mixed breastfeeding and early weaning to be added to the multiple models.

**Table 3:** Prevalence of health care actions performed in different periods for the promotion and success of breastfeeding. Natal/Brazil, 2019.

Variables	Yes	No
	
	n (%)	n (%)
Prenatal		
Number of consultations ≥6	304 (73.6)	109 (26.4)
Guidance on breastfeeding	244 (59.5)	166 (40.5)
Delivery room		
Skin-to-skin contact	246 (59.3)	169 (40.7)
Breastfeeding in the first hour	242 (58.5)	172 (41.5)
Rooming-in setting		
Guidance on breastfeeding	338 (81.4)	77 (18.6)
Guidance on expressing breast milk	244 (58.8)	171 (41.2)
Guidance on breast care	310 (74.7)	105 (25.3)
Guidance for not offering other milk	226 (54.5)	189 (45.5)
Guidance for not offering a pacifier	213 (51.3)	202 (48.7)
Guidance for not offering a baby bottle	211 (50.8)	204 (49.2)
Puerperium		
Home visit	107 (31.2)	236 (68.8)
Guidance on breastfeeding during home visits	79 (73.8)	28 (26.2)
Newborn consultation	145 (41.9)	201 (58.1)

Source: research data (2019).

Multivariate analysis (Table 4) showed a significant association for the offer of mixed breastfeeding and early weaning throughout the neonatal period. In the first 48 hours, maternal age ≤ 20 years was a protective factor of mixed breastfeeding (RR = 0.64; 95%CI: 0.47–0.86). Conversely, primiparous mothers (RR = 1.37; 95%CI: 1.11–1.60) and whose delivery was cesarean section (RR = 1.20; 95%CI: 1.00–1.45) showed a higher risk for the offer of infant formula. At 7 days, the use of pacifier, with a 3.21 times greater risk (95%CI: 1.63–6.32), and lack of paternal support during breastfeeding, with a 4.98 times greater risk (95%CI: 2.54–9.79), remained in the adjusted model. At 28 days, only the use of pacifier remained, with a 2.48 times greater risk for the early weaning with infant formula, cow’s milk or porridge (95%CI: 1.53–4.02).

**Table 4:** Relative risk of factors associated with the introduction of complements to breast milk and early weaning in the neonatal period. Natal/Brazil, 2019.

Variables	2 days		7 days		28 days	
		
	Gross RR (95%CI)	Adjusted RR (95%CI)	Gross RR (95%CI)	Adjusted RR (95%CI)	Gross RR (95%CI)	Adjusted RR (95%CI)
Maternal characteristics						
Maternal age ≤ 20 years 20 to 29 years > 30 years	0.77 (0.59–1.01) 0.76 (0.62–0.92)	0.64 (0.47–0.86) 0.70 (0.57–0.87)	-	-	-	-
Education in years > 9 years Up to 9 years	-	-	1 1.82 (0.92–3.61)	-	-	-
Family income^a^ > 1 minimum salary ≤ 1 minimum salary	-	-	1 0,56 (0.28–1.10)	-	-	-
Diabetes No Yes	1 1.24 (0.98–1.58)	-	1 2.17 (0.99–4.76)	-	-	-
Parity Multiparous Primiparous	1 1.17 (0.97–1.41)	1 1.37 (1.11-1.60)	1 1.73 (0.88–3.43)	-	1 1.69 (1.05–2.72)	-
Type of delivery Vaginal Cesarean section	1 1.25 (1.04–1.51)	1 1.20 (1.00–1.45)	-	-	1 1.39 (0.86–2.24)	-
Characteristics of NB						
Birth weight 2,500–4,000g > 4,000g	1 1.32 (0.99–1.71)	-	-	-	1 0.43 (0.11–1.65)	-
Care characteristics						
Companion during delivery Yes No	1 1,.7 (0.96–1.42)	-	-	-	1 1.56 (0.95–2.54)	-
Breast feeding in the 1^st^ hour of life. Yes No	-	-	1 1.93 (0.96–3.89)	-	-	-
Paternal support to breast feeding Yes No	-	-	1 4.4 (2.25–8.64)	1 4.98 (2.54–9.79)	1 1.84 (1.10–3.08)	-
Use of pacifier No Yes	-	-	1 2.67 (1.37–5.16)	1 3.21 (1.63–6.32)	1 2.48 (1.53–4.02)	1 2.48 (1.53–4.02)
Offer of complements No Yes	-		1 2.13 (1.00–4.53)	-	-	-

NB: newborn; 95%CI: 95% confidence interval; RR: Relative Risk.

^a^ Minimum salary = R$ 998.00.

Multiple Poisson regression.

## DISCUSSION

This study has identified health care failures in the promotion, protection and support of EBF in the first 28 days of life of newborn in the four participating maternity hospitals. Although EBF expressed a low prevalence in the first 48 hours, there was a considerable increase at the end of the neonatal period, whose rates were higher than those reported by Carreiro et al., with 1,608 records of mother and children assisted in a clinic specialized in breastfeeding from 2004 to 2016^15^.

Early weaning and introduction of other foods in an unsafe manner can contribute to neonatal and infant morbidity and mortality^5^. Conversely, the discontinuity of breastfeeding can be conditioned not only by biological factors, but also socioeconomic and health care. The pertinent literature points out that the lack of incentive on the part of health care institutions, the lack of family support for breastfeeding women and the offer of pacifiers are among the main causes of early weaning^7,16^.

This study found that, as substitute and/or complement to breast milk, infant formula and cow’s milk were offered. In maternity hospitals, due to the shortage of pasteurized milk in the milk bank, infant formula was offered at a suitable dilution. After hospital discharge, at home, the surveyed mothers replaced or complemented breast milk with infant formula or whole cow’s milk or porridge, whose dilution was not suitably performed by some of these mothers. Thus, it was not meeting the guidelines specified on the product labels, which could undermine the food security and nutritional status in a critical period of development, where the child already has vulnerable health conditions.

Despite the increase in the prevalence of EBF at 7 days, the offer of porridge – made with cow’s milk, flour and sugar – drew our attention. This type of food is not suitable for children, mainly in the neonatal phase; the high amount of sugar and protein concentration can impair the kidney function and the microbiota of newborn, in addition to having high inflammatory and allergenic potential from a genetic determination^12^. From the first week, water was offered to almost all newborn who received the complement, which may be due to the lack of knowledge or misunderstanding. Nevertheless, when in EBF or using infant formula, such an offer is not necessary, given that the water content of breast milk and the suitably reconstituted formula already meet the child’s needs. Water supply may promote a deceptive satiety and reduce the breast stimulation and the number of feedings^2^.

In the first 48 hours, still in the maternity hospital, the maternal age behaved as a protective factor in favor of EBF, which differs from the study lead by Bentley et al., in Australia, with 24,713 mother-infant in the period of 2010–2013^17^. Primiparity and cesarean delivery were risk factors to mixed breastfeeding, in line with other studies^15,18^. According to the WHO, the aforementioned factors represent vulnerability to maternal and child health. This may explain, for example, the possible greater professional assistance to breastfeeding women in the first days postpartum, with respect to age. Nevertheless, after hospital discharge, maternal age did not behave as a protection factor to early weaning. Regarding to primiparity, the systematic review performed by Garrison and collaborators^19^ justifies that, in this condition, the inexperience in breastfeeding contributes to offer formula supplementation to the newborn early due to maternal immaturity to deal with the difficulties normally present in the first days of breastfeeding.

As for the type of delivery, studies indicate that caesarean section can delay lactogenesis or milk flow. For this reason, it is usually a predisposing factor to the professional indication for mixed breastfeeding due to shortage or absence of colostrum^20,21^. Although this was the main indication for the use of the complement at 48 hours of birth in this study, it is not justified by BFHI. Among the justifiable reasons, we can mention the maternal physiological conditions (characteristics of the breast that lead to difficulty in latching and sucking, as cracked nipples and mastitis, for example) and the newborn conditions (such as hypoglycemia and oral-motor disorder)^22^.

When considering that colostrum has a high nutritional, immunological and protective contribution to the intestinal microbiome, it is fundamental that it be offered to the neonate^23^. The pertinent literature points out that skin-to-skin contact and breastfeeding in the first hour of life favor not only its offer, but also prolonged EBF and, above all, the mother-child bond. Accordingly, these practices should be encouraged by health professionals and family members^24^. We should also underline that the use of the complement while still in the maternity hospital, when not suitably prescribed, can influence their stay after hospital discharge, being associated with early weaning^11^.

Nevertheless, in this study, after the use of the complement was adjusted to other variables, such as the case of maternal characteristics, it was not predictive for the remainder of the neonatal period. Besides, we should emphasize that the lack of medical records about the reasons for indicating the complement, observed in this study, warns of the need for a more careful professional assistance to the binomial, in order to achieve the effective promotion of EBF, thus respecting the BFHI principles – substantially, the step six of the “Ten Steps to Successful Breastfeeding”, which says *“Do not give the newborn baby any food or drink other than breast milk, unless such procedure has a medical indication”*^8^.

In this approach, the first week of comprehensive health, which is instituted by the *Política Nacional de Atenção Integral à Saúde da Criança* (PNAISC – National Policy for Comprehensive Child Health Care), is based on health protection, health promotion and prevention of ill-health^25^. Home visits allow longitudinal health care and surveillance, are also essential for encouraging the act of breastfeeding^27^. At home, health professionals identify risk situations in the binomial and guide the mother both in self-care and in newborn care. Nevertheless, this study identified that most parturient women were not visited; and, when the visit happened, almost 30% of them did not receive guidelines on breastfeeding. These findings are opposed to those reported in the study of meta-analyses by Cheng et al.^27^ that evaluated the efficacy of home-based intervention with professional support, which showed benefits of home visits in relation to EBF.

The weaknesses in the visits revealed in this study emphasize that the meetings may be insufficient in number and content to support the continuity of breastfeeding. The mother’s insecurity about the suitable quantity of milk produced to meet the baby’s nutritional needs was the main reason for the discontinuity of breastfeeding after hospital discharge. The reference to “little milk” may be related to cultural beliefs that characterize breast milk as “weak”, since, given the persistence of crying, it would not be sufficient to satisfy newborn. This finding corroborates with the studies by Oliveira et al (2017)^28^, and reinforce the need for an approach that promotes a better understanding of these concepts and values. The WHO emphasizes that external support for breastfeeding (professional, family or friends) influences the duration of this practice, being even essential when newborn is in EBF^1^. Such support can be exercised either by verbal encouragement and physical support during breastfeeding moments or helping the mother with domestic activities and caring for other children (whenever required). This support improves coexistence and minimizes stress to the breastfeeding woman – which, naturally, results from biological and psychological problems of both mother and child –, common to this phase.

After the inclusion of maternal and health care variables, only the absence of paternal support and the use of pacifier remained as risk factors for early weaning. When the partner supports the breastfeeding, it increases the chances of breastfeeding in the first hour of life, and avoids early weaning. In our study, the absence of paternal support increased in almost 5 times the risk of early weaning, and this result corroborated with the studies of Muelbert and Gilgliani, which were carried out with 207 mothers in the city of Porto Alegre - Brazil, from 2006 to 2008^29^.

The use of pacifier was risk factor to early weaning at 7 and 28 days, corroborating with a cohort which was conducted for Gasparin et al.^7^. We found similar results in the literature review that was performed by Batista and collaborators from 2010 to 2015, with 150 pairs mother/infant in the first month^14^. The lack of knowledge about the harms, the preconception, the lack of family support, the post-surgical discomfort in the puerperium and, especially, the desire to nurture the baby can influence this offer. The pacifier is not recommended by the MH and this is explained in the step nine of the “Ten Steps to Successful Breastfeeding”, which says “*Do not provide artificial nipples or pacifiers to breastfed children*”^8^. The *Sociedade Brasileira de Pediatria* (SBP – Brazilian Society of Pediatrics) also contraindicates this use since pacifiers affect breathing and, above all, the development of sucking and swallowing, thus undermining breastfeeding^30^.

This study was limited to the identification of feeding practices in the neonatal period and associated risk factors to the mixed breastfeeding and early weaning, thus the reasons that led mothers to offer baby bottles and pacifiers were not assessed. The adopted methodology (including the training of the research team) and the sample size of this study strengthen the results found. Despite the sample losses, the telephone call may have favored the high number of participants in the subsequent periods, since it made contact possible, even though they lived in distant locations. The follow-up contributed to mitigate memory biases, once the mothers were called immediately after the studied periods.

Early weaning with the offer of infant formula in the first 48 hours of life of newborn, still in the maternity hospital, was present in more than half of the sample. At 7 and 28 days at home, cow’s milk, porridge and water were added. The primiparity, the cesarean delivery, the absence of paternal support and the use of pacifier behaved as risk factors for early weaning. These findings emphasize the need for more robust assistance to the puerperium – a phase of high vulnerability for the mother-child binomial –, including greater family and, above all, professional involvement.

We concluded that the precariousness found, both in maternity hospitals and in home visits, reflect the importance of strengthening health care actions to achieve the prolonged breastfeeding recommended by MH. This should be consolidated at all levels of care and at all stages of the binomial (prenatal, delivery, birth and puerperium) in the first 1.000 days, which is the most critical phase that influences child’s health and development.

## References

[1] World Health Organization. Guideline: protecting, promoting and supporting breastfeeding in facilities providing maternity and newborn services. Geneva (CH): WHO; 2017. 29565522

[2] Ministério da Saúde (BR), Secretaria de Atenção Primária à Saúde. Departamento de Promoção à Saúde. Guia alimentar para crianças brasileiras menores de dois anos. Brasília, DF; 2019.

[3] Nações Unidas Brasil. UNICEF: apenas 40% das crianças no mundo recebem amamentação exclusiva no início da vida. Brasília, DF; 2019 [cited 2020 May 22]. Available from: https://brasil.un.org/pt-br/83869-unicef-apenas-40-das-criancas-no-mundo-recebem amamentacao-exclusiva-no-inicio-da-vida

[4] Boccolini CS, Boccolini PMM, Monteiro FR, Venâncio SI, Giugliani ERJ. Breastfeeding indicators trends in Brazil for three decades. Rev Saude Publica. 2017;51:108. 10.11606/S1518-8787.2017051000029 PMC5697916 29166437

[5] Victora CG, Bahl R, Barros AJD, França GVA, Horton S, Krasevec J, et al. Breastfeeding in the 21st century: epidemiology, mechanisms, and lifelong effect. Lancet 2016;387(10017):475-90. 10.1016/S0140-6736(15)01024-7 26869575

[6] Universidade Federal do Rio de Janeiro. Estudo Nacional de Alimentação e Nutrição Infantil ENANI-2019: resultados preliminares: indicadores de aleitamento materno no Brasil. Rio de Janeiro, RJ: UFRJ; 2020 [cited 2020 Set 10]. Available from: https://enani.nutricao.ufrj.br/wp-content/uploads/2020/08/Relatorio-preliminar-AM-Site.pdf

[7] Gasparin VA, Strada JKR, Moraes BA, Betti T, Gonçalves AC, Espírito Santo LC. Pairs seen by lactation consultants and cessation of exclusive breastfeeding in the first month. Rev Esc Enferm USP. 2019;53:e03422. 10.1590/s1980-220x2018010003422 30673052

[8] World Health Organization; UNICEF. Baby-friendly Hospital Initiative: ten steps to successful breastfeeding. Geneva: WHO; UNICEF; 2018 [cited 2020 May 10]. Available from: https://www.who.int/activities/promoting-baby-friendly-hospitals/ten-steps-to-successful-breastfeeding

[9] Ministério da Saúde (BR), Secretaria de Atenção à Saúde. Estratégia Nacional para Promoção do Aleitamento Materno e Alimentação Complementar Saudável no Sistema Único de Saúde: manual de implementação. Brasília, DF; 2015.

[10] Brasil. Lei Nº 11265, de 3 de janeiro de 2006. Regulamenta a comercialização de alimentos para lactentes e crianças de primeira infância e também a de produtos de puericultura correlatos. Diário Oficial da União. 4 jan 2006; Seção 1:1-3.

[11] Chantry CJ, Dewey KG, Peerson JM, Wagner EA, Nommsen-Rivers LA. In-hospital formula use increases early breastfeeding cessation among first-time mothers intending to exclusively breastfeed. J Pediatr. 2014;164(6):1339-45.e45. 10.1016/j.jpeds.2013.12.035 PMC4120190 24529621

[12] Kelly E, DunnGalvin G, Murphy BP, Hourihane JO. Formula supplementation remains a risk for cow’s milk allergy in breast-fed infants. Pediatr Allergy Immunol. 2019;30(8):810-6. 10.1111/pai.13108 31297890

[13] Ministério da Saúde (BR), Secretaria de Ciência, Tecnologia e Insumos Estratégicos, Departamento de Ciência e Tecnologia. Avaliação da atenção ao pré-natal, ao parto e aos menores de um ano na Amazônia Legal e Nordeste, Brasil, 2010 Brasília, DF: MS; 2013 [cited 2020 Dec 7]. Available from: http://bvsms.saude.gov.br/bvs/publicacoes/livro_avaliacao_da_atencao_ao_pre-natal_web.pdf

[14] Batista CLC, Ribeiro VS, Nascimento MDSB. Influência do uso de chupetas e mamadeiras na prática do aleitamento materno. J Heath Biol Sci. 2017;5(2):184-91. 10.12662/2317-3076jhbs.v5i2.1153.p184-191.2017

[15] Carreiro JA, Francisco AA, Abrão ACFV, Marcacine KO, Abuchaim ESV, Coca KP. Dificuldades relacionadas ao aleitamento materno: análise de um serviço especializado em amamentação. Acta Paul Enferm. 2018;31(4):430-8. 10.1590/1982-0194201800060

[16] Wagner LPB, Mazza VA, Souza SRRK, Chiesa A, Lacerda MR, Soares L. Strengthening and weakening factors for breastfeeding from the perspective of the nursing mother and her family. Rev Esc Enferm USP. 2020;54:e03563. 10.1590/s1980-220x2018034303564 32401890

[17] Bentley JP, Nassar N, Porter M, Vroome M, Yip E, Ampt AJ. Formula supplementation in hospital and subsequent feeding at discharge among women who intended to exclusively breastfeed: an administrative data retrospective cohort study. Birth. 2017;44(4):352-62. https://doi:10.1111/birt.12300 10.1111/birt.12300 28737234

[18] Andrade HS, Pessoa RA, Donizete LCV. Fatores relacionados ao desmame precoce do aleitamento materno. Rev Bras Med Fam Comunidade. 2018;13(40):1-11. 10.5712/rbmfc13(40)1698

[19] Garrison MP, Maisano P. Systematic review of factors influencing non-medically indicated formula supplementation of newborns in the hospital setting. Nurs Womens Health. 2019;23(4):340-50. https://doi:10.1016/j.nwh.2019.06.003 10.1016/j.nwh.2019.06.003 31400848

[20] Rocha BO. Hipogalactia inicial, fatores de risco para o desmame precoce e promoção do aleitamento materno em primíparas atendidas em um Hospital Amigo da Criança no Brasil [dissertação]. Belo Horizonte, MG: Universidade Federal de Minas Gerais; 2018.

[21] Pinheiro JMF, Menêzes TB, Brito KMF, Melo ANL, Queiroz DJM, Sureira TM. Prevalência e fatores associados à prescrição/solicitação de suplementação alimentar em recém-nascidos. Rev Nutr. 2016; 29(3):367-75. 10.1590/1678-98652016000300007

[22] World Health Organization; UNICEF. Acceptable medical reasons for use of breast-milk substitutes. Geneva (CH): WHO; UNICEF; 2009. 24809113

[23] Tsopmo A. Phytochemicals in human milk and their potential antioxidative protection antioxidants. Antioxidants (Basel). 2018;7(2):32. 10.3390/antiox7020032 PMC5836022 29470421

[24] Moore ER, Bergman N, Anderson GC, Medley N. Early skin-to-skin contact for mothers and their healthy newborn infants. Cochrane Database Syst Rev. 2016;11(11):CD003519. 10.1002/14651858.CD003519.pub4 PMC6464366 27885658

[25] Ministério da Saúde (BR, Secretaria de Atenção à Saúde, Departamento de Ações Programáticas e Estratégicas. Política Nacional de Atenção Integral à Saúde da Criança: orientações para implementação. Brasília, DF; 2018.

[26] Organización Mundial de la Salud; Fondo de las Naciones Unidas para la Infancia. Visitas domiciliarias al recién nacido: una estratégia para aumentar la supervivencia: declaración conjunta OMS/UNICEF. Ginebra (CH): OMS; 2009.

[27] Cheng LY, Wang X, Mo KP. The effect of home-based intervention with professional support on promoting breastfeeding: a systematic review. Int J Public Health. 2019;64(7):999‐1014. 10.1007/s00038-019-01266-5 31197407

[28] Oliveira AKP, Melo RA, Maciel LP, Tavares AK, Amando AR, Sena CRS. Práticas e crenças populares associadas ao desmame precoce. Rev Enferm. 2017;35(3):303-12. 10.15446/av.enferm.v35n3.62542

[29] Muelbert M, Giugliani ERJ. Factors associated with the maintenance of breastfeeding for 6, 12, and 24 months in adolescent mothers. BMC Public Health. 2018;18(1):675. 10.1186/s12889-018-5585-4 PMC5984453 29855364

[30] Sociedade Brasileira de Pediatria, Departamento Científico de Aleitamento Materno. Guia Prático de Atualização. Uso de Chupeta em crianças amamentadas: prós e contras. Rio de Janeiro: SBP; 2017; 3:1-16. [cited 2020 May10]. Available from: https://www.sbp.com.br/fileadmin/user_upload/Aleitamento-Chupeta_em_Criancas_Amamentadas.pdf

